# Freeze-Dried Curdlan/Whey Protein Isolate-Based Biomaterial as Promising Scaffold for Matrix-Associated Autologous Chondrocyte Transplantation—A Pilot In-Vitro Study

**DOI:** 10.3390/cells11020282

**Published:** 2022-01-14

**Authors:** Katarzyna Klimek, Marta Tarczynska, Wieslaw Truszkiewicz, Krzysztof Gaweda, Timothy E. L. Douglas, Grazyna Ginalska

**Affiliations:** 1 Chair and Department of Biochemistry and Biotechnology, Medical University of Lublin, Chodzki 1 Street, 20-093 Lublin, Poland; wieslaw.truszkiewicz@umlub.pl (W.T.); g.ginalska@umlub.pl (G.G.); 2 Department and Clinic of Orthopaedics and Traumatology, Medical University of Lublin, Jaczewskiego 8 Street, 20-090 Lublin, Poland; marta.tarczynska-osiniak@umlub.pl (M.T.); krzysztof.gaweda@umlub.pl (K.G.); 3 Engineering Department, Lancaster University, Gillow Avenue, Lancaster LA 1 4YW, UK; t.douglas@lancaster.ac.uk; 4 Materials Science Institute (MSI), Lancaster University, Lancaster LA 1 4YW, UK

**Keywords:** arthroscopy, cartilage, cell culture, chondrocyte isolation, curdlan, β-1,3-glucan, knee, MACI, MACT, implantation

## Abstract

The purpose of this pilot study was to establish whether a novel freeze-dried curdlan/whey protein isolate-based biomaterial may be taken into consideration as a potential scaffold for matrix-associated autologous chondrocyte transplantation. For this reason, this biomaterial was initially characterized by the visualization of its micro- and macrostructures as well as evaluation of its mechanical stability, and its ability to undergo enzymatic degradation in vitro. Subsequently, the cytocompatibility of the biomaterial towards human chondrocytes (isolated from an orthopaedic patient) was assessed. It was demonstrated that the novel freeze-dried curdlan/whey protein isolate-based biomaterial possessed a porous structure and a Young’s modulus close to those of the superficial and middle zones of cartilage. It also exhibited controllable degradability in collagenase II solution over nine weeks. Most importantly, this biomaterial supported the viability and proliferation of human chondrocytes, which maintained their characteristic phenotype. Moreover, quantitative reverse transcription PCR analysis and confocal microscope observations revealed that the biomaterial may protect chondrocytes from dedifferentiation towards fibroblast-like cells during 12-day culture. Thus, in conclusion, this pilot study demonstrated that novel freeze-dried curdlan/whey protein isolate-based biomaterial may be considered as a potential scaffold for matrix-associated autologous chondrocyte transplantation.

## 1. Introduction

Cartilage damage is very common in orthopaedic patients, most often involving knees but also other joints such as, hips, ankles, and elbows. Such damage often results from improperly performed physical exercise, disease and trauma. Moreover, the risk of cartilage damage significantly increases with the age of the patient [1,2]. Minor cartilage damage, involving its surface layer (grades I and II according to Outerbridge scale), is usually treated pharmacologically. However, more serious lesions (grade III and IV, Outerbridge scale), which involve cracks in the deep cartilage layer, require surgical intervention [3,4,5].

One of the promising approaches to support cartilage regeneration is autologous chondrocyte implantation (ACI). Nevertheless, this technique possesses serious drawbacks. For instance, its medical effectiveness is limited to small-area lesions. Moreover, in the course of this method, primary chondrocytes are first isolated and then cultured on polystyrene (i.e., in two-dimensional conditions), which leads to their dedifferentiation towards fibroblast-like cells. It was demonstrated that such cells possess limited ability to regenerate hyaline articular cartilage [6,7,8,9,10,11]. In the case of large-area lesions, there is a need to use grafts which will replace the fragments of missing tissue. Although autografts are considered as a “gold standard” in regeneration of cartilage defects, their availability is highly limited. For these reasons, current medical interest focuses on synthetic biomaterials, which not only have a composition and a structure similar to those of natural tissue, but also exhibit similar physicochemical, mechanical, and biological properties [6,7,8,9,10,11]. The biomaterials may be implanted directly without cells or in combination with cells; this constitutes a modern therapeutic strategy called matrix-associated autologous chondrocyte transplantation (MACT) [12,13,14,15]. The MACT procedure may be divided into three general stages. In the first step, the patient undergoes arthroscopy, which allows for cartilage biopsy. Then, harvested tissue is transported to the laboratory where it is subjected to enzymatic digestion in order to isolate primary chondrocytes. After a few days of cell culture in vitro, a suitable number of cells is seeded directly on the scaffold. Cells on the biomaterial are further cultivated under two-dimensional (2D) or three-dimensional (3D) conditions for several days to weeks. Finally, the living graft is transplanted into the patient’s knee during open surgery (miniarthrotomy) [16,17]. To date, several MACT products have been allowed onto the European medical devices market. For instance, Hyalograft^®^ C (Fidia Advanced Biomaterials, Turin, Italy) is a hyaluronan-based scaffold seeded with chondrocytes. Before implantation, this product is incubated for at least two weeks under 2D conditions [18]. Matrix-associated autologous chondrocyte implantation, i.e., MACI^®^ (Genzyme, Boston, MA, USA) is a product that includes chondrocytes grown on membrane composed of collagen type I and III. Such a construct is maintained for one week in 3D culture. In turn, NOVOCART 3D (TETEC, Reutlingen, Germany) is composed of collagen type I and chondroitin sulphate. Chondrocytes are seeded directly on this bilayered sponge and then maintained for two days in 3D culture. Most importantly, many short- and long-term clinical follow-ups confirmed that application of MACT products significantly accelerates regeneration of cartilage defects in orthopaedic patients [12,16,19,20,21,22,23,24,25].

The aim of this study was to determine whether a novel freeze-dried curdlan/whey protein isolate-based biomaterial may be considered as a promising scaffold for matrix-associated autologous chondrocyte transplantation. For this reason, a curdlan/whey protein isolate-based biomaterial was fabricated and its structural, mechanical, and biological properties were evaluated in vitro. Thus, the macro- and microstructure of the biomaterial were evaluated using stereoscopic microscopy and scanning electron microscopy, respectively. The Young’s modulus of the scaffold was assessed based on mechanical tests. Moreover, in vitro biodegradation of the novel scaffold was estimated during 9-week incubation in collagenase type II solution. Importantly, in-vitro cytocompatibility of the novel scaffold was evaluated using primary human chondrocytes. To the best of our knowledge, this is the first study in which a curdlan/whey protein isolate-based biomaterial was fabricated and evaluated as a potential MACT product.

## 2. Materials and Methods

### 2.1. Fabrication of Freeze-Dried Curdlan/Whey Protein Isolate-Based Scaffold

This biomaterial was prepared according to the procedure described in Polish patent application no. P.437236 entitled “Biomaterial based on β-1,3-glucan (curdlan) for regeneration of cartilage tissue and/or bone and method of its production”. Briefly, an aqueous solution of 30 wt.% whey protein isolate (WPI) (BiPRO, Davisco Foods International, Agropur Cooperative, Eden Prairie, MN, USA) was prepared, and then 1 mL of this solution was added to 0.08 g of curdlan powder (80 kDa, WAKO pure Chemicals Industries, Osaka, Japan). After thorough mixing, the homogeneous solution was heated at 90 °C for 15 min (Fixed Dry Block Heater, BTD, Grant Instruments, Beaver Falls, PA, USA). After cooling to room temperature, the biomaterial was cut into suitable samples (approx. 8 mm in diameter and 2 mm in height for most of the experiments or approx. 8 mm in diameter and 8 mm in height for mechanical testing). Next, the specimens were frozen (−80 °C, 2 days, New Brunswick^TM^ Innova^®^ U101, Eppendrof, Warsaw, Poland) and then freeze-dried (24 h, LYO GT2-Basic, SRK Systemtechnik GmbH, Riedstadt, Germany). At the end, samples were sterilized with ethylene oxide. For further experiments, the biomaterial will hereafter be denoted as “Cur_WPI”.

### 2.2. Macro- and Microstructural Characterization

The macrostructure of biomaterial was characterized using a stereoscopic microscope (Olympus SZ61TR, Olympus, Poland). In turn, the microstructure of biomaterial was visualized by a field emission gun scanning electron microscope (FEG-SEM, JSM-7800F, Joel Ltd., Tokyo, Japan), using a lower secondary electron detector. Firstly, a sample was mounted on standard aluminium pin stubs using double-sided conductive carbon-adhesive dots. Afterwards, its surface was coated with approx. 5 nm of gold (at 20 mA for 60 s, 1 × 10^−2^ mBar, under argon) using a Gold Sputter Coater (Q150RES, Quorum Technologies Ltd., East Sussex, UK).

### 2.3. Evaluation of Mechanical Properties

Compression tests were performed using an INSTRON 3345 testing machine (Instron^®^, Norwood, MA, USA) with a 500-N load cell. All samples were compressed at a basic load rate of 5 mm/min until the maximum strain value of 50% was reached. The following values were measured: displacement (mm), force (kN), and time. Subsequently, the compressive stress (σ), compressive strain (ε), and consequentially the sample’s Young’s modulus (E) were calculated. To produce reliable results, five individual specimens were used (*n* = 5).

The Young’s modulus was obtained from the gradient between 0% and 10% on a compressive stress and compressive strain graphs. This particular interval was used as it showed the most linear results. An average was then calculated from each sample. The compressive stress used to represent each specimen was obtained from calculating the mean values of the compressive stress at 5% compressive strain.

### 2.4. Evaluation of Susceptibility of Biomaterial to Enzymatic Degradation

Before the experiment, 0.02% collagenase II (Worthington Biochemical Corporation, Lakewood, NJ, USA) solution in phosphate-buffered saline (PBS, Sigma-Aldrich, Chemicals, Warsaw, Poland) was prepared. The solution was sterilized using a 0.22-μm syringe filter (Bionovo^®^, Legnica, Poland). Then, four separate samples (*n =* 4) of the biomaterial (of comparable weight) were placed in sterile 15-mL conical tubes, and 5 mL of collagenase II solution was added to each tube. Additionally, samples soaked only in 5 mL PBS were used as an experimental control. The tubes were placed into an incubator (37 °C, 50 rpm, New Brunswick^TM^ Innova^®^ 42 Incubator Shakers, Eppendrof, Warsaw, Poland). The experiment was carried out for 9 weeks, and collagenase II or PBS solutions were replaced by new portions every 3 weeks. After 3, 6, and 9 weeks of the experiment, the samples were removed from tubes, rinsed with PBS, frozen (−80 °C, 2 days, New Brunswick^TM^ Innova^®^ U101, Eppendrof, Warsaw, Poland), and then freeze-dried (24 h, LYO GT2-Basic, SRK Systemtechnik GmbH, Riedstadt, Germany). Subsequently, the samples were weighed. The biodegradation in vitro of samples was assessed by loss of their weight using the following Equation (1):

(1) $$Degradation\;\left(\%\right)=\frac{M0-Mt}{M0}\times 100\%$$

where *M*0 denotes initial biomaterial weight and *Mt* denotes biomaterial weight after 3, 6, and 9 weeks of the experiment, respectively.

### 2.5. Evaluation of Chondrocyte–Biomaterial Interactions In Vitro

#### 2.5.1. Isolation and Identification of Primary Human Chondrocytes

The primary human chondrocytes’ isolation was carried out according to an optimized procedure developed by the first author, based on previously available protocols [26,27,28]. Human cartilages were harvested during knee arthroscopy after obtaining consent of the Bioethics Committee of Medical University of Lublin, Poland (approval no. KE-0254/114/2020 from June 2020). The patients gave their written informed consent for their biological material to be used for research purposes. For this study, the cartilage tissue samples were obtained from four patients in order to obtain a mixture of cells from various donors. Immediately after the biopsy, the tissues were placed in sterile PBS solution and transferred to the laboratory. Then, digestion solution, i.e., 0.2% collagenase II (Worthington Biochemical Corporation, Lakewood, NJ, USA) solution in culture medium—Dulbecco’s modified eagle’s medium/F12 (DMEM/F12 1:1, Gibco, ThermoFisher Scientific, Waltham, MA, USA) supplemented with 15% foetal bovine solution (FBS, Pan-Biotech, Aidenbach, Germany), and 0.1% antibiotic antimycotic solution (Sigma-Aldrich Chemicals, Warsaw, Poland) was prepared. The solution was sterilized using a 0.22-μm syringe filter. The human cartilage tissue samples were placed in a plastic Petri dish, rinsed three times with PBS solution, and weighed (the total mass was 2.32 g). Then, the tissues were cut into small pieces using a sterile scalpel, PBS solution was removed, and digestion solution was added (proportion: 10 mL of digestion solution per 1 g of cartilage). A Petri dish containing the cartilage pieces (obtained from four donors) in digestion solution was placed into an incubator (37 °C, 16 h, 50 rpm, MS Hybridization Shaking Oven, Major Science, Saratoga, CA, USA). Afterwards, the obtained cell suspension was filtered through a 70 μm cell strainer into a sterile 50 mL conical tube and centrifuged at 300× *g* for 10 min. (Sigma 3-18 K, POLYGEN, Gliwice, Poland). At the same time, complete culture medium was prepared, i.e., DMEM/F12 1:1 medium (Gibco, ThermoFisher Scientific, Waltham, MA, USA) supplemented with 10% FBS (Pan-Biotech, Aidenbach, Germany), 10 ng/mL human fibroblast growth factor 2 (hFGF-2, R&D SYSTEMS, Canada, USA), 1 ng/mL human transforming growth factor β-1 (hTGF-β1, R&D SYSTEMS, Canada, USA), and antibiotics (10 U/mL penicillin, 10 μg/mL streptomycin, Sigma-Aldrich Chemicals, Warsaw, Poland). The cell pellet was resuspended in culture medium and centrifuged. This step was repeated three times. Cell viability and number was assessed using an automated cell counter (Countless 3 FL, ThermoFisher Scientific, Waltham, MA, USA). Then, approx. 5 × 10^6^ cells in culture medium were added to the cell culture flasks at a concentration of 5 × 10^4^ cells/cm^2^ and incubated at 37 °C in a humidified atmosphere (Heraeus Cytoperm 2, Thermo Fisher Scientific, Waltham, MA, USA). In turn, approx. 1 × 10^6^ cells were subjected to real-time quantitative PCR (RT-qPCR) in order to evaluate cell phenotype. Before RT-qPCR, the cells were centrifuged at 600× *g* for 10 min. (Sigma 3-18 K, POLYGEN, Gliwice, Poland). Then, total RNA was extracted using NucleoSpin RNA kit (Macherey-Nagel, Düren, Germany), and its concentration and its purity were determined using a UV spectrophotometer (Synergy H4 hybrid reader, BioTek, Winooski, VT, USA). Subsequently, 24 ng of total RNA was used for the one-step RT-qPCR SYBR Green assay (One-step NZY RT-qPCR Green kit, Nzytech, Lisbon, Portugal). The reaction was carried out using a LightCycler 480 II (Roche, Rotkreuz, Switzerland). The RT-qPCR was performed with the use of the following parameters: 20 min. at 50 °C (reverse transcription), followed by 10 min. at 95 °C, 40 cycles for 15 s. at 95 °C (denaturation), and 30 s. at 61 °C (annealing/extension). Glyceraldehyde-3-phosphate dehydrogenase (*GAPDH*) was applied as a housekeeping gene. The qPCR primers were supplied by Sigma-Aldrich (Warsaw, Poland) and are summarized in Table 1. The analysis was performed in triplicate. Relative gene expressions of collagen type I (*COL1A1*), collagen type II (*COL2A1*), aggrecan (*ACAN*), and SRY-box transcription factor 9 (*SOX-9*) were calculated via the 2^−∆∆Ct^ method [29]. Data were expressed as mean values of three replicates.

**Figure 1:**
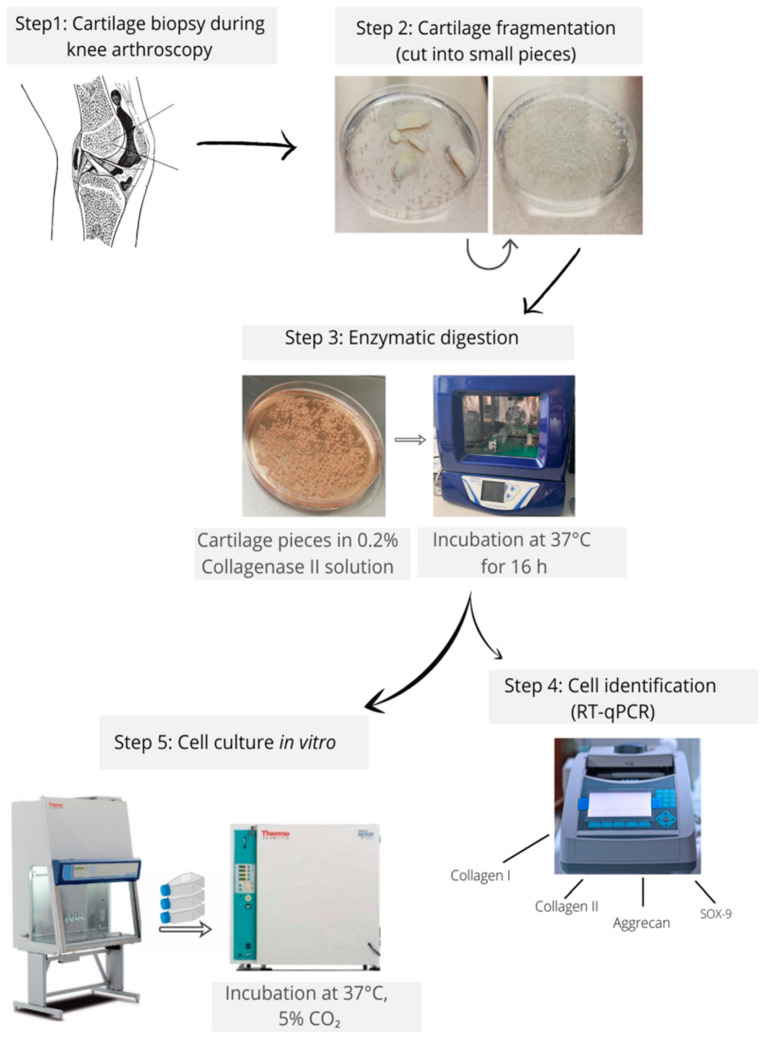
General procedure of isolation and identification of primary human chondrocytes.

The general procedure of isolation and identification of primary human chondrocytes is presented in Figure 1.

#### 2.5.2. Assessment of Chondrocyte Viability

Before the experiment, the biomaterials were soaked in complete culture medium for 12 h at 37 °C. Then, chondrocytes (passage 1) in complete culture medium were seeded directly on the scaffold surface at a concentration of 2 × 10^5^ cells/sample. The chondrocytes seeded on polystyrene (PS) at the same concentration were considered as control cells. After 48-h incubation at 37 °C in a humidified atmosphere, the cell viability was assessed qualitatively using a Live/Dead Cell Double Staining Kit (Sigma-Aldrich, Warsaw, Poland). The cells were observed under a confocal laser scanning microscope (CLSM, Olympus Fluoview equipped with FV1000, Shinjuku, Japan). Green or red fluorescence was emitted by live or dead cells, respectively.

#### 2.5.3. Assessment of Chondrocyte Proliferation

Before the experiment, the biomaterials were soaked in complete culture medium for 12 h at 37 °C. Then, chondrocytes (passage 1) in complete culture medium were seeded directly on three separate scaffold samples (*n* = 3) at a concentration of 1 × 10^5^ cells/sample. The chondrocytes seeded on polystyrene (PS) (*n* = 3) at the same concentration were considered as control cells. The experiment was conducted for 12 days at 37 °C in a humidified atmosphere. The cell medium was replaced every two days. After 4-, 8-, and 12-day incubation, the cell proliferation was assessed quantitatively as well as qualitatively. Thus, a Cell Counting Kit-8 (WST-8, Sigma-Aldrich, Warsaw, Poland) was used to determine the metabolic activity of cells, which is proportional to their number. In turn, a Hoechst 33342 (Sigma-Aldrich, Warsaw, Poland) and AlexaFluor^TM^ 635 Phalloidin (Invitrogen, ThermoFisher Scientific, Waltham, MA, USA) were employed in order to visualize cell nuclei and cytoskeleton, respectively. The cells were observed under a CLSM (Olympus Fluoview equipped with FV1000, Shinjuku, Japan) and the nuclei emitted a blue fluorescence, while the cytoskeleton gave a red one.

#### 2.5.4. Assessment of Expression of Cartilage-Specific Genes

Before analysis, the cells were seeded directly on the three separate scaffold specimens (*n* = 3) and three PS samples (*n* = 3) as described in Section 2.5.3. “Assessment ofChondrocyte Proliferation”. After 12 days of culture, the RT-qPCR was used to evaluate the expression level for *COL2A1*, *ACAN*, and *SOX-9*. Additionally, expression of *COL1A1* was evaluated in order to determine chondrocyte dedifferentiation towards fibroblast-like cells. The analysis was performed according to the procedure described in detail above (Section 2.5.1. “Isolation and identification of primary human chondrocytes”). The data were normalized to the expression levels in cells cultured on PS for the same length of time (for 12 days).

#### 2.5.5. Microscope Observations of Cartilage-Related Markers

The cells were cultured on Cur_WPI and PS as described above (Section 2.5.4). After 12 days of culture, collagen type I, collagen type II, aggrecan, and SOX-9 were visualized via immunofluorescence staining. Thus, the cells were rinsed with PBS, fixed with 3.7% paraformaldehyde, permeabilized with 0.2% Triton^TM^X-100, and blocked with 1% bovine serum albumin solution (all reagents from Sigma-Aldrich, Warsaw, Poland). Then, the cells were stained overnight with 10 μg/mL of the following primary antibodies: rabbit polyclonal anti-collagen I antibody (Invitrogen, ThermoFisher Scientific, Waltham, MA, USA), rabbit polyclonal anti-collagen II antibody (Abcam, Cambridge, UK), mouse monoclonal anti-aggrecan antibody (Invitrogen, ThermoFisher Scientific, Waltham, MA, USA), and rabbit monoclonal anti-SOX-9 antibody (Abcam, Cambridge, UK). Subsequently, the cells were stained with 2 μg/mL of secondary antibodies, namely goat anti-rabbit IgG (H + L) antibody-conjugated with AlexaFluor^®^ 488 or goat anti-mouse IgG (H + L) antibody-conjugated with AlexaFluor^®^ 488 (both reagents from Abcam, Cambridge, UK). Additionally, cell nuclei were stained with Hoechst 33342 (Sigma-Aldrich, Warsaw, Poland). The cells were observed using a CLSM (Olympus Fluoview equipped with FV1000, Shinjuku, Japan). Nuclei emitted a blue fluorescence, while evaluated markers emitted a green one.

### 2.6. Statistical Analysis

The results were presented as mean values ± standard deviation (SD). The statistical analysis was performed using a one-way ANOVA test, followed by a Tukey’s multiple comparison test. The differences between tested groups were considered as statistically significant when *p* < 0.05 (GraphPad Prism 5, Version 5.04 Software).

## 3. Results and Discussion

### 3.1. Macro- and Microstructures of Scaffold

**Figure 2:**
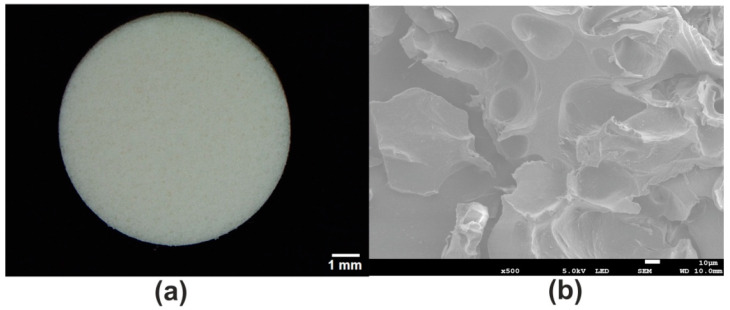
Stereoscopic microscopy (**a**) and SEM (**b**) images showing macro- and microstructures of the novel Cur_WPI scaffold. The magnification and scale bar of the SEM image were 500× and 10 μm, respectively.

The stereoscopic microscope revealed that novel Cur_WPI scaffold possessed a porous structure (Figure 2a). This observation was also confirmed by SEM (Figure 2b). Such a phenomenon is most likely associated with the technique applied to fabricate the biomaterial. It is known that a combination of freezing and freeze-drying allows porous biomaterials to be obtained [32,33]. Porous, 3D biomaterials, thanks to high specific surface area, constitute appropriate scaffolds for cell cultivation [34]. Thus, it seems that the surface of Cur_WPI biomaterial should support cell adhesion and proliferation.

### 3.2. Mechanical Properties of Scaffold

Compression tests demonstrated that the Cur_WPI biomaterial possessed good mechanical properties for its potential application, i.e., as a scaffold for cartilage regeneration (Table 2). It was established that the value of Young’s modulus of cartilage increases from the superficial to the deep zone and ranges from 0.08 to 6.44 MPa [35,36]. Thus, it indicates that the Cur_WPI scaffold should be the most suitable for regeneration of superficial and middle zones of cartilage. Nevertheless, it was worth underlining that the value of Young’s modulus determined for the Cur_WPI biomaterial was satisfactory, when compared with data presented by other researchers. For instance, Nanda et al. developed collagen-based scaffolds for cartilage tissue engineering applications and showed that their Young’s moduli were close to 0.15 MPa [37]. In turn, Rogan et al. fabricated gelatine-based hydrogels, which accelerated cartilage regeneration in vivo [38]. The Young’s modulus of these biomaterials was equal to 0.33 MPa.

### 3.3. Enzymatic Biodegradation of Scaffold

**Figure 3:**
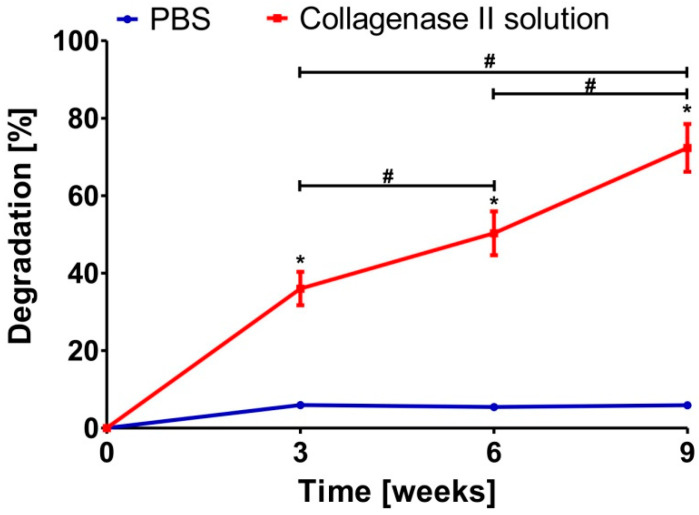
Degradation percentage of Cur_WPI biomaterial after 9-week incubation in PBS and 0.02% collagenase II solution. * Significantly different results compared with data obtained in PBS solution; ^#^ significantly different results between data obtained in 0.02% collagenase solution after different time of incubation; one-way ANOVA test followed by Tukey’s multiple comparison, *p* < 0.05.

The biodegradation assay revealed that Cur_WPI biomaterial was stable in PBS solution for the whole duration of the experiment (Figure 3). Over 9 weeks, only a slight decrease in biomaterial mass (approx. to 5.8%) was observed. In turn, a significant decrease in weight of the Cur_WPI biomaterial was noted when it was placed in collagenase II solution (Figure 3). After 3, 6, and 9 weeks of incubation, the degradation percentages were close to 36.02 ± 4.32%, 50.30 ± 5.66%, and 72.34 ± 6.17%, respectively. The ability to undergo biodegradation is a very important feature of implantable polymer-based biomaterials. It was found that protein-based biomaterials most often undergo biodegradation too rapidly [39,40,41]. Polysaccharide-based biomaterials degrade more slowly, while scaffolds composed of synthetic polymers degrade very slowly or not at all [41,42,43]. Thus, fabrication of biomaterials composed of proteins and polysaccharides allow the production of scaffolds with the ability to degrade in a controlled manner [44]. This assumption was confirmed in this study. The obtained Cur_WPI scaffold was characterized by its controllable rate of degradation (gradual, non-immediate) over 9 weeks. From a medical point of view, this phenomenon is very favourable, because after implantation, the Cur_WPI biomaterial should degrade at a suitable rate, correlated with cell proliferation and tissue regeneration [45].

### 3.4. Characterization of Primary Human Chondrocytes

**Figure 4:**
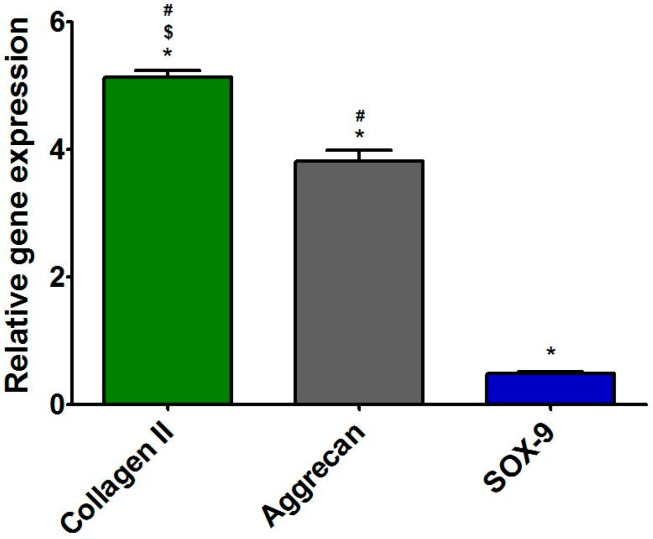
Relative expression level of genes: collagen II, aggrecan, and SOX-9 in freshly isolated chondrocytes. The results were normalized to the expression level of collagen I in isolated cells. * Significantly different results compared with expression level of collagen I; ^$^ significantly different results compared with expression level of aggrecan; ^#^ significantly different results compared with expression level of SOX-9; one-way ANOVA test followed by Tukey’s multiple comparison, *p* < 0.05.

After isolation, the cells were subjected to RT-qPCR analysis in order to assess the expression level of characteristic cartilage-specific genes, i.e., *COL2A1*, *ACAN*, and *SOX-9*. Moreover, the expression level of *COL1A1* was also evaluated to exclude the presence of dedifferentiated cells. The RT-qPCR analysis (Figure 4) demonstrated that the level of *COL2A1* and aggrecan expression in cells was approx. five and four times higher when compared with the expression level of *COL1A1*. Thus, these results indicated that the isolated cells were chondrocytes instead of fibroblasts. After isolation, the cells were cultured on polystyrene only for 3 days to restrict their dedifferentiation towards fibroblast-like cells [6,46,47]. Then, cells were detached and seeded directly on Cur_WPI scaffolds and on polystyrene (control). Afterwards, cell viability (Section 3.5.1), cell proliferation (Section 3.5.2), and the presence of cartilage-specific markers (Section 3.5.2) were evaluated.

### 3.5. Cytocompatibility of Scaffold

#### 3.5.1. Cell Viability

**Figure 5:**
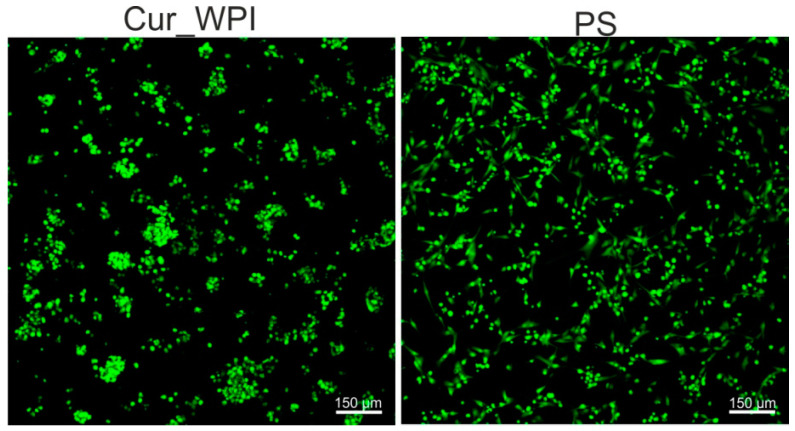
Confocal microscope images showing viability of chondrocytes cultured on Cur_WPI biomaterial and polystyrene (PS) after 48-h incubation. Live cells emitted green fluorescence, while dead cells gave red fluorescence. Magnification 100×, scale bar was 150 μm.

The first step of cell culture experiments involved the assessment of chondrocyte viability. After 48-h incubation, live/dead staining showed that cell grown on both Cur_WPI scaffold and on polystyrene (PS) were live (green fluorescence), and no dead cells were present (red fluorescence) (Figure 5). This observation indicated that Cur_WPI scaffold as well as PS supported chondrocyte adhesion and growth. Most importantly, chondrocytes cultured on the Cur_WPI biomaterial were round and grew in characteristic cell clusters, which suggests that the fabricated scaffold allows them to preserve their characteristic chondrocyte phenotype. In turn, the cells cultured on PS were not only round, but also flattened, which indicates that dedifferentiation of chondrocytes towards fibroblast-like cells had started.

#### 3.5.2. Cell Proliferation

**Figure 6:**
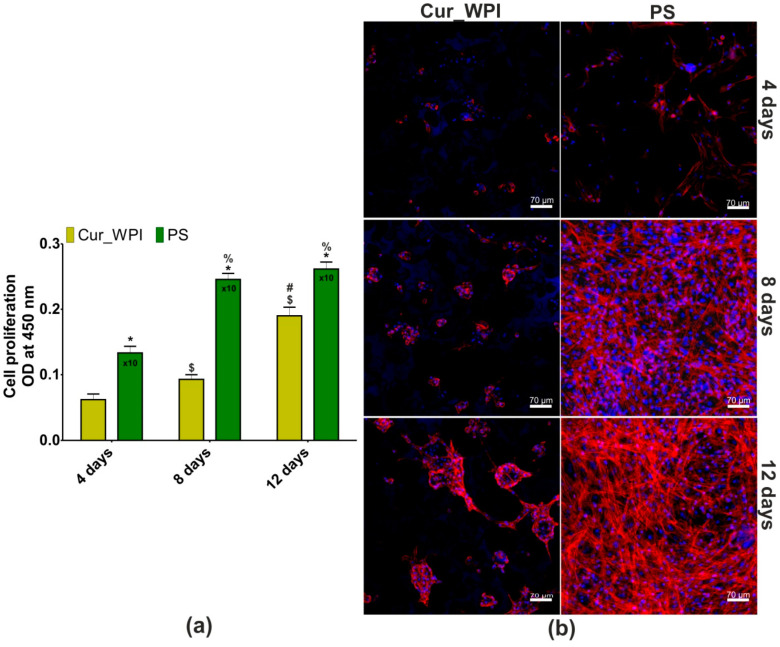
Proliferation of chondrocytes after 4, 8, and 12 days of culture (**a**). The results were obtained via a WST-8 assay (* significantly different results between Cur_WPI biomaterial and polystyrene (PS) at the same time of incubation; ^$^ significantly different results compared with Cur_WPI biomaterial at day 4; ^#^ significantly different results compared with Cur_WPI biomaterial at day 8; ^%^ significantly different results compared with PS at day 4; one-way ANOVA test followed by Tukey’s multiple comparison, *p* < 0.05). Confocal microscope images showing morphology of chondrocytes cultured on Cur_WPI biomaterial and polystyrene (PS, control) after 4-, 8-, and 12-day incubation (**b**). Nuclei emitted blue fluorescence (visible blue fluorescence in the structure of biomaterial was emitted by WPI), while F-actin filaments gave red fluorescence; magnification 200×, scale bar equals 70 μm.

The WST-8 assay revealed that metabolic activity of chondrocytes cultured both on Cur_WPI scaffolds and polystyrene (PS) increased with the duration of the experiment (Figure 6a). Although the metabolic activity of cells cultured on PS was higher compared with the metabolic activity of chondrocytes grown on Cur_WPI, this phenomenon was to be expected, as cell adaptation to the 3D environment (on biomaterials) is usually slower compared with the 2D environment (on flat polystyrene) [48]. Most importantly, the metabolic activity of chondrocytes cultured on Cur_WPI increased at a statistically significant rate (*p* < 0.05), which proves that this biomaterial supports cell proliferation. The results obtained during the WST-8 test were confirmed by confocal microscope observations (Figure 6b). The higher number of cells was observed with increased time of incubation. The chondrocytes seeded on Cur_WPI scaffold grew in characteristic cell clusters, while the cells cultured on PS were flattened and well-spread, indicating their dedifferentiation towards fibroblast-like cells. These observations are in good agreement with results obtained by other researchers [49,50,51,52,53,54]. For instance, Homicz et al. [49] demonstrated that chondrocytes cultured on polystyrene possessed elongated and spindle-like shapes, while chondrocytes that grew on alginate-based biomaterial had a spherical morphology. Similarly, Malda et al. [50] showed that chondrocytes which grew on polystyrene had a fibroblast-like morphology, but those cultured in Cytodex-1 microcarriers preserved their characteristic phenotype. Wang et al. [53] indicated that 2D culture of chondrocytes (on polystyrene) led to their dedifferentiation towards fibroblast-like cells. In turn, 3D culturing (on silk-based scaffold) of dedifferentiated chondrocytes ensured their redifferentiation. Thus, it is worth noting that Cur_WPI scaffold not only promotes chondrocyte divisions but also allows them to maintain their characteristic morphology. These properties are crucial for biomaterials intended for matrix-associated autologous chondrocyte transplantation [9].

#### 3.5.3. Presence of Cartilage-Specific Markers—RT-qPCR Analysis and Immunofluorescence Staining

**Figure 7:**
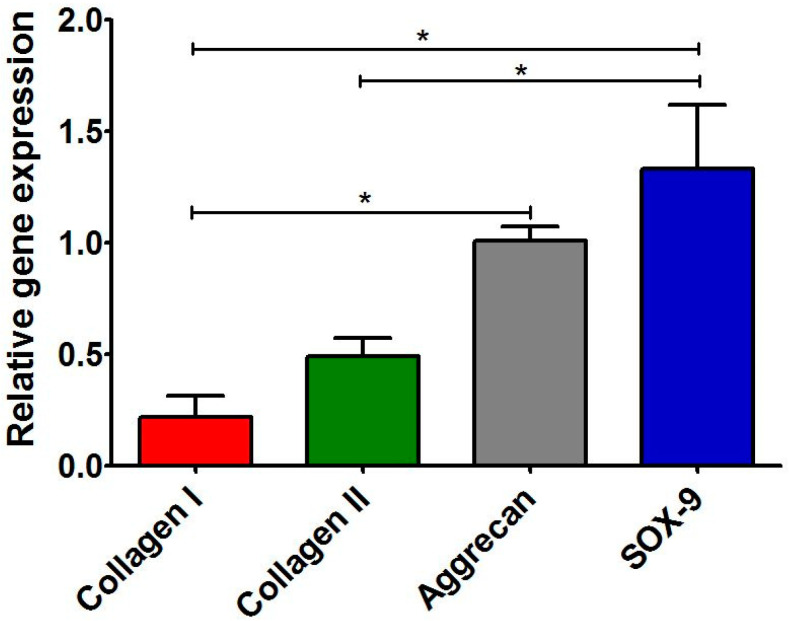
Relative expression of genes: collagen I, collagen II, aggrecan, and SOX-9 in chondrocytes cultured on Cur_WPI scaffold for 12 days. The results were normalized to expression levels of genes in cells cultured on polystyrene. * Significantly different results between expression level of evaluated genes; one-way ANOVA test followed by Tukey’s multiple comparison, *p* < 0.05.

After 12 days of incubation, the RT-qPCR analysis showed that chondrocytes cultured on Cur_WPI biomaterial expressed the highest amount of *SOX-9* and *ACAN* (Figure 7). Surprisingly, the expression of *COL2A1* in the cells was approx. two and three times lower, when compared with the expression of *SOX-9* and *ACAN*, respectively. Although, chondrocytes cultured on biomaterial expressed greater (but not statistically significantly higher) amounts of *COL2A1* than *COL1A1*, their expressions were lower than those in cells grew on PS. Thus, expression of two important cartilage-specific genes (*SOX-9* and *ACAN*) in chondrocytes cultured on Cur_WPI scaffold was greater than in cells cultured on PS, and at the same time, the expression of gene responsible for chondrocyte dedifferentiation towards fibroblast-like cells (*COL1A1*) was suppressed.

**Figure 8:**
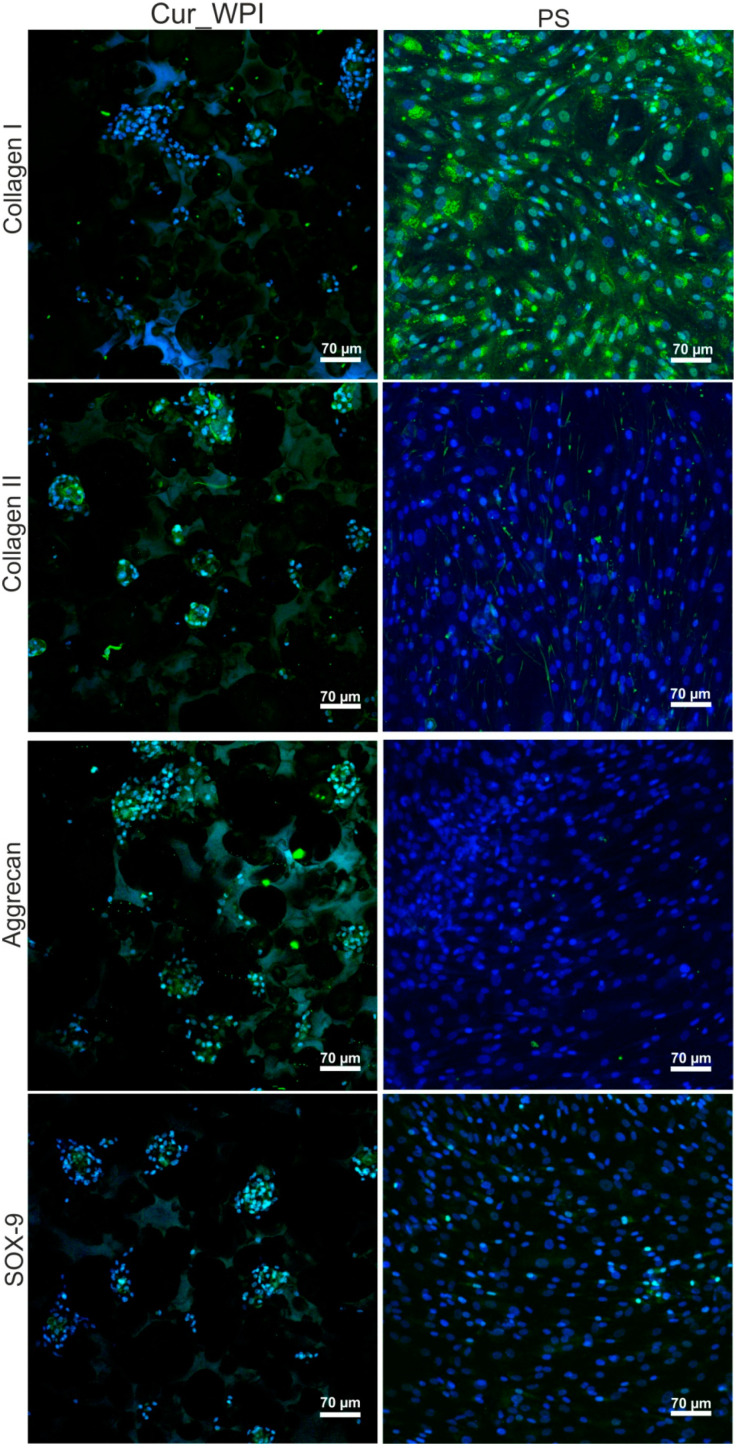
Confocal microscope images presenting characteristic markers—collagen II, aggrecan, and SOX-9 after the 12-day culturing of chondrocytes on Cur_WPI biomaterials and polystyrene (PS). Collagen I was also visualized to determine chondrocyte dedifferentiation towards fibroblast-like cells. Nuclei emitted blue fluorescence, while evaluated markers gave green fluorescence (visible green fluorescence in the structure of biomaterial was emitted by WPI); magnification 200×, scale bar equals 70 μm.

The confocal microscope observation (Figure 8) proved that cells cultured on polystyrene (PS) underwent dedifferentiation towards fibroblast-like cells. Thus, intensive immunofluorescence of collagen I in cells was observed. Collagen II and aggrecan were not detected, while only very slight immunofluorescence of SOX-9 was visible. In the case of cells grown on Cur_WPI scaffolds, immunofluorescence staining (Figure 8) partially confirmed the results obtained during RT-qPCR analysis (Figure 7). It was observed that the cells synthesized collagen II, aggrecan, and SOX-9. In turn, the immunofluorescence of collagen I in cells was very weak. These results confirm data obtained by other researchers. It is well known that, unlike 2D cultures, 3D culturing of chondrocytes allows the maintenance of their phenotype. The cells growing on biomaterials synthesize extracellular matrix (ECM), which is abundant in collagen II and aggrecan. Moreover, they express chondrocyte-specific factors, primarily SOX-9. SOX-9 expression is specific for chondrocyte differentiation [49,50,52,54,55,56,57,58,59,60]. Thus, based on confocal microscope observations, it seems that Cur_WPI biomaterial should allow preservation of typical chondrocyte phenotype over 12 days of culture. Nevertheless, in order to confirm this phenomenon precisely, additional experiments (i.e., ELISA test, Western blot analysis) with the use of chondrocytes obtained from higher number of independent patients will be performed in the future.

## 4. Conclusions

In this pilot study, a novel freeze-dried curdlan/whey protein isolate-based biomaterial was fabricated and assessed in the context of its potential application, i.e., as a scaffold for matrix-associated autologous chondrocyte transplantation. Ideally, scaffolds designed for cartilage tissue engineering should provide a 3D template for chondrocyte growth. For this reason, they should be porous, mechanically stable, biodegradable, biocompatible, and should promote new tissue formation. It was demonstrated that the Cur_WPI biomaterial possessed a porous structure and was characterized by a suitable value of Young’s modulus, which was close to those of the superficial and middle zones of cartilage. It also exhibited degradability in a controlled manner for 9 weeks. Most importantly, the Cur_WPI biomaterial supported human chondrocyte viability and proliferation, while maintaining their characteristic phenotype. This scaffold also promoted synthesis of cartilage-specific markers (collagen type II, aggrecan, and SOX-9) by the chondrocytes. Thus, the developed Cur_WPI biomaterial, thanks to its physicochemical properties as well as cytocompatibility, and ability to prevent dedifferentiation of chondrocytes towards fibroblast-like cells, seems to be a promising scaffold for matrix-associated autologous chondrocyte transplantation. Nevertheless, for precise characterization of its biomedical potential, additional in-vitro analysis (with the use of chondrocytes obtained from higher number of patients) and in vivo study will be performed in future.

## 5. Patents

The Cur_WPI biomaterial was prepared according to the procedure described in the Polish patent application no. P.437236 entitled “Biomaterial based on β-1,3-glucan (curdlan) for regeneration of cartilage tissue and/or bone and method of its production”—Katarzyna Klimek, Grazyna Ginalska, Marta Tarczynska, Krzysztof Gaweda.

## Figures and Tables

**Table 1:** List of primers for RT-qPCR analysis. The primer sequences for collagen type II and aggrecan were developed based on literature data [30], while primer sequences for collagen type I, SRY-box transcription factor 9, and glyceraldehydes-3-phosphate were developed using Primer-BLAST tool that is available online on the website of National Center for Biotechnology Information (NCBI) [31].

Gene	Primer Sequence (5′–3′)	Product Size (bp)
collagen type I (*COL1A1*)	F: GGCCCAGAAGAACTGGTACA R: AATCCATCGGTCATGCTCTC	81
collagen type II (*COL2A1*)	F: GGCAATAGCAGGTTCACGTACA R: CGATAACAGTCTTGCCCCACTTA	79
aggrecan (*ACAN*)	F: AGCCTGCGCTCCAATGACT R: TAATGGAACACGATGCCTTTCA	107
SRY-box transcription factor 9 (*SOX-9*)	F: GAGACTTCTGAACGAGAGCGA R: CGTTCTTCACCGACTTCCTCC	125
glyceraldehyde-3-Phosphate dehydrogenase (*GAPDH*)	F: CACCACACTGAATCTCCCCT R: TGGTTGAGCACAGGGTACTT	115

**Table 2:** The Young’s modulus value of curdlan/whey protein isolate-based biomaterial.

Young’s Modulus (MPa) ± SD
0.849 ± 0.157

## Data Availability

Data available on reasonable request. The data may be obtained from Katarzyna Klimek.
